# Cancer-associated fibroblasts, and clinicopathological characteristics and prognosis of gastric cancer: A systematic review and meta-analysis

**DOI:** 10.3389/fonc.2023.1048922

**Published:** 2023-02-17

**Authors:** Jinwu Wei, Mingxia Wang, Guixiang Li

**Affiliations:** ^1^ The Second Clinical Medical College, Lanzhou University, Lanzhou, Gansu, China; ^2^ Cancer Center, Lanzhou University Second Hospital, Lanzhou, Gansu, China

**Keywords:** cancer-associated fibroblasts, gastric cancer, clinicopathological characteristics, prognosis, meta-analysis

## Abstract

**Objective:**

To systematically evaluate the relationship between cancer-associated fibroblasts (CAFs) and clinicopathological characteristics and prognosis of gastric cancer, so as to provide new directions and clinical evidence for the diagnosis and treatment of this disease.

**Methods:**

We searched PubMed, Embase, Web of Science, and The Cochrane Library to identify studies on the correlation between tumor-associated fibroblasts and the diagnosis and prognosis of gastric cancer. Two researchers screened the literature independently to extract data, evaluated the quality of the included studies, and used the Review Manager 5.4 software to perform a meta-analysis.

**Results:**

A total of 14 studies involving a total of 2,703 patients were included. The meta-analysis results showed that high expression of CAFs was associated with stage III–IV gastric cancer (relative risk ratio [RR]=1.59; 95% confidence interval [CI]: [1.24–2.04]; P=0.0003), lymph node metastasis (RR=1.51; 95% CI: [1.23–1.87]; P=0.0001), serosal infiltration (RR=1.56, 95% CI: [1.24–1.95]; P=0.0001), diffuse and mixed types in Lauren classification (RR=1.43; 95% CI: [1.18–1.74]; P=0.0003), vascular invasion (RR=1.99; 95% CI: [1.26–3.14]; P=0.003), and overall survival (hazard ratio [HR]=1.38; 95% CI: [1.22–1.56]; P<0.00001). However, the high expression of CAFs was not significantly correlated with poorly differentiated gastric cancer (RR=1.03; 95% CI: [0.96–1.10]; P=0.45) and gastric cancer with tumor diameter >5 cm (RR=1.34; 95% CI: [0.98–1.83]; P=0.07).

**Conclusion:**

The findings of this meta-analysis demonstrated that high expression of CAFs is closely associated with the traditional pathological indicators related to poor prognosis in gastric cancer, and is a valuable prognostic factor in this setting.

**Systematic Review Registration:**

https://www.crd.york.ac.uk/PROSPERO/, identifier CRD42022358165.

## 1 Introduction

Gastric cancer (GC) is a type of malignant tumors with the fifth and fourth highest incidence and mortality rate worldwide, respectively (1). Owing to advances in treatment, the incidence and mortality of GC have decreased in recent years. Nevertheless, this cancer continues to profoundly affect the lives of individuals in East Asia, particularly in China (2). Nowadays, due to extensive screening, as well as the availability of endoscopic or surgical treatment, the overall survival (OS) of patients with GC has been greatly prolonged. However, many patients are diagnosed at a late stage and have poor prognosis. The main treatments for advanced GC include chemotherapy, targeted therapy, and immunotherapy (3). The progress achieved in these treatments is inseparable from the in-depth study of the clinicopathological characteristics of GC. Some biomarkers have been found, such as microsatellite instability, mismatch repair deficient, human epidermal growth factor receptor 2 (HER2), programmed cell death ligand 1 (PD-L1), tumor mutation burden, and Epstein–Barr virus. These biomarkers are linked to the prognosis of GC and play a great role in the molecular typing and treatment of this disease (4).

In addition to targeting the tumor tissue itself, the environment of tumor growth, also termed the tumor microenvironment (TME), has been receiving considerable research attention. Cancer-associated fibroblasts (CAFs) are defined as cells that exist in the stroma of tumors without epithelial, endothelial, or leukocyte markers; they are elongated in shape and do not carry oncogene mutations (5). Studies suggested that CAFs specifically express α-smooth muscle actin (α-SMA) and fibroblast-activation protein (FAP). Hence, CAFs can be identified through their morphology and these cell markers (6). As an important part of the TME, CAFs are directly related to tumor growth, invasion, metastasis, and therapeutic effect (7).

It has been shown that CAF expression is associated with the prognosis of some types of cancer (8, 9). Is high expression of CAFs an efficient biomarker for the differentiation of patients with GC who are at high risk of diagnosis at an advanced stage and poor prognosis? In this meta-analysis, we integrated existing studies to further investigate the relationship between CAFs and the clinicopathological characteristics and prognosis of patients with GC to provide new directions and clinical evidence for molecular typing and targeted therapy of GC.

## 2 Materials and methods

This study adhered to the PRISMA guidelines (10) for meta-analyses and systematic reviews. This analysis was based on data from previously studies registered on PROSPERO (CRD42022358165).

### 2.1 Eligibility criteria

The inclusion criteria were as follows (1): patients diagnosed with GC based on histopathology (2); expression of CAFs detected by immunohistochemistry, using α-SMA and FAP as markers of CAFs; and (3) studies including available data on one or more of the following clinicopathological characteristics: clinical stage, differentiation, tumor depth, lymph node metastasis, Lauren classification, tumor size, vascular invasion, and OS. The exclusion criteria were (1): reviews, letters, meetings, abstracts, unavailability of full text; and (2) literature written in a language other than English.

### 2.2 Search strategy

PubMed, Embase, Web of Science, and The Cochrane Library were searched for studies on CAFs in GC from the establishment of the database until September 1, 2022. The retrieval was carried out by combining free words with subject terms, and the final retrieval formula was determined by multiple pre-retrieval. The search terms included: CAFs, tumor-associated fibroblasts, stomach neoblasts, GC, etc. The specific search strategy is shown in the **Supplementary File**. Other eligible studies were retrieved from the references cited in the selected articles and relevant literature.

### 2.3 Literature screening and data extraction

Two researchers (J.W. and M.W.) independently screened the literature according to the inclusion and exclusion criteria and, subsequently, extracted and cross-checked the data. In case of disagreement, consensus was reached following discussion with a third investigator. The extracted data included publication information (e.g., title, first author, publication time, country), subjects (e.g., sample size, age, markers used for CAF detection, CAF expression determination standard), clinicopathological data (e.g., tumor size, stage, grade, lymph node metastasis, tumor depth, vascular invasion, Lauren classification, follow-up time, follow-up rate, OS, study design scheme, quality). If it was not possible to obtain the original data from the literature, the corresponding author was contacted; in case of no response, data were measured and extracted from the relevant images.

### 2.4 Quality assessment

The Newcastle–Ottawa Quality Assessment Scale (NOS) was independently used by two researchers to evaluate the risk of bias of the included studies (11). In case of disagreement, consensus was reached following discussion with a third investigator. The highest NOS score is 9, with scores >6 denoting high-quality research.

### 2.5 Statistical analysis

The Review Manager 5.4 software was used for the meta-analysis of data included in this study. For continuous and dichotomous data, mean difference and relative risk ratio (RR) were used as the effect size, respectively. Point estimates and 95% confidence intervals (95% CI) were provided for each effect size. The chi-squared test and P-values were used to qualitatively analyze the statistical heterogeneity among the results, and I^2^ was used to quantitatively analyze the heterogeneity. When I^2^ ≤ 50% or I^2^>50%, a fixed effect model or random effect model was used for the meta-analysis, respectively. P-values <0.05 denoted statistically significant differences. The meta-analysis was also analyzed by Stata/SE 16. Publication bias was assessed visually with a funnel plot and the Egger weighted regression statistic, with P<0.05 indicating significant publication bias.

## 3 Results

### 3.1 Search results

**Figure 1:**
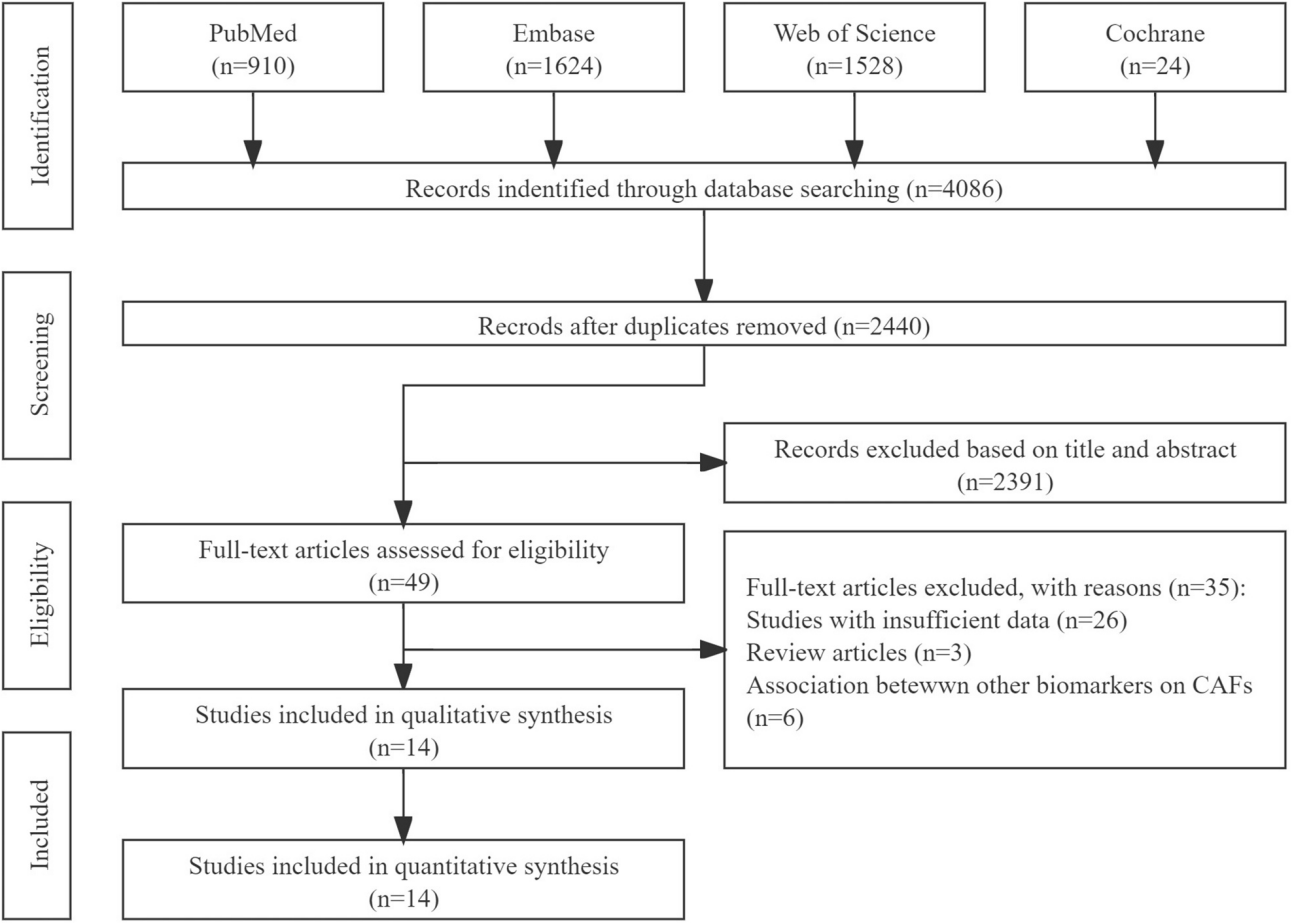
PRISMA flow diagram for study selection. CAF, cancer-associated fibroblast.

According to our search strategy, a total of 4,086 studies were identified. After removing duplicate articles, 2,440 studies were selected. By reading the titles and abstracts, we removed 2,391 records because of non-relevance with the theme. After reviewing the full texts of the 46 potentially eligible records in detail, the following studies were excluded: studies with insufficient data (n=26); reviews (n=3); and studies investigating associations between other biomarkers on CAFs (n=6). Eventually, 14 studies (12–25) were included in this meta-analysis. The selection process is illustrated in **Figure 1**.

### 3.2 Study characteristics

These 14 studies, involving a total of 2,703 patients, were included in the pooled analysis. Ten and four studies were conducted in China (12–15, 17–20, 22, 23) and Japan (16, 21, 24, 25), respectively, with publication dates spanning from 2007 to 2020. All studies examined more than one clinicopathological characteristics of GC. OS was recorded in eight studies. Study quality, assessed by the NOS score, ranged from 7 to 8. The characteristics and NOS score of the included studies are shown in **Table 1**.

**Table 1:** Characteristics of studies included in the meta-analysis.

First author	Year	Country	No.	Age of patients	CAF biomarker	Determination of CAF expression in samples	Preoperative treatment	Follow-up time	Follow-up rate	Outcome	NOS
(reference number)				(years)				(months)			score
Chao Lin (12)	2016	China	387	NA	α-SMA	Proportion of positive cells stained by IHC	NA	79	100%	②③④⑤⑧	8
								Median: 49			
Hu Song (13)	2017	China	112	Mean: 64	FAP	Staining positive area and expression score by IHC	NA	59	NA	①②③④⑧	7
								Median:42			
Junli Zhang (14)	2020	China	227	Range: 31–98	α-SMA	Average number of positive cells by IHC	No neoadjuvant	Range: 3.2–99.5	100%	①②⑤⑥	8
							therapy	Median: 36.7			
Kangkang Zhi (15)	2010	China	100	NA	α-SMA	Positive staining area and staining intensity by IHC	No neoadjuvant therapy	NA	NA	①②③④⑥	7
Kenji Kuroda (16)	2019	Japan	584	NA	α-SMA	Percentage of stained positive cells and staining intensity by IHC	NA	60	100%	①②③④⑥⑦⑧	8
Lihui Shan (17)	2012	China	60	Range: 21–65	FAP	Relative percentage of positive staining areas and staining intensity by IHC	No neoadjuvant therapy	NA	NA	①②④⑤	8
Liming Gao (18)	2017	China	110	Mean: 57	FAP	Degree of staining and the proportion of stained positive cells by IHC	No neoadjuvant therapy	NA	NA	①②③④⑥	7
Ruifen Wang (19)	2013	China	60	Range: 21–72	FAP	Relative percentage of positive staining areas by IHC	No neoadjuvant therapy	NA	NA	①②③④⑤	7
Shenghua Zhan (20)	2020	China	268	Range: 33–88	α-SMA	Proportion of positive cells stained by IHC	NA	60	73.88% (198/268)	①②③④⑥⑧	7
Taizan Minam (21)	2019	Japan	120	Range: 38–87	α-SMA	Degree of staining by IHC	NA	128	NA	①③④⑦⑧	7
								Median: 51			
Xiliang Cong (22)	2020	China	215	NA	α-SMA	Percentage of stained positive cells and staining intensity by IHC	No neoadjuvant therapy	Median: 41.4	91.16% (196/215)	①②⑥	8
Yongchen Ma (23)	2018	China	95	NA	FAP	Percentage of stained positive cells and staining intensity by IHC	No neoadjuvant therapy	60	76.84% (73/95)	①②③④⑧	8
Youji Fukumoto (24)	2009	Japan	100	Range: 33–93	FAP	Quantitative analysis of the number and proportion of positive cells by IHC	NA	180	100%	②③⑦⑧	7
								Median: 39			
Yuhiko Fuyuhiro (25)	2010	Japan	265	NA	α-SMA	Semi-quantitative analysis of the expression levels and proportion of positive cells by IHC	NA	177	88.30% (234/265)	②③④⑦⑧	7
								Median: 58			

α-SMA, alpha-smooth muscle actin; CAF, cancer-associated fibroblast; FAP, fibroblast activation protein; IHC, immunohistochemical; NA, not available; No., number of patients; NOS, Newcastle–Ottawa Quality Assessment Scale; ① clinical stage; ② differentiation; ③ lymph node metastasis; ④ tumor depth; ⑤ Lauren classification; ⑥ tumor size; ⑦ vascular invasion; ⑧ overall survival.

### 3.3 Correlation between CAFs and clinicopathological characteristics

#### 3.3.1 High expression of CAFs was associated with stage III–IV GC

**Figure 2:**
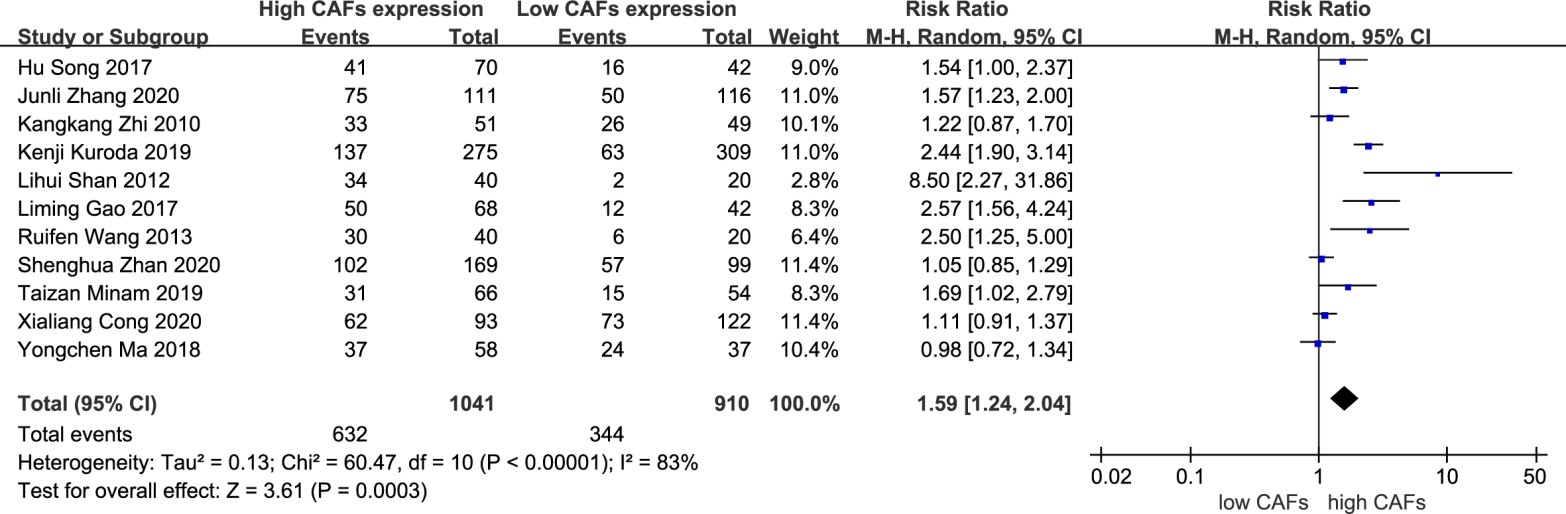
High expression of CAFs is associated with stage III–IV gastric cancer. CAF, cancer-associated fibroblast; CI, confidence interval.

A total of 11 studies (13–23) were included, involving 1,951 patients. The meta-analysis results showed that patients with high expression of CAFs were at a significantly increased risk of progressing into stage III–IV GC. The rate of stage III–IV GC in samples with high and low expression of CAFs was 60.7% and 37.8%, respectively. The RR of the study was 1.59 (95% CI: 1.24–2.04; P=0.0003) (**Figure 2**).

#### 3.3.2 High expression of CAFs was associated with lymph node metastasis in GC

**Figure 3:**
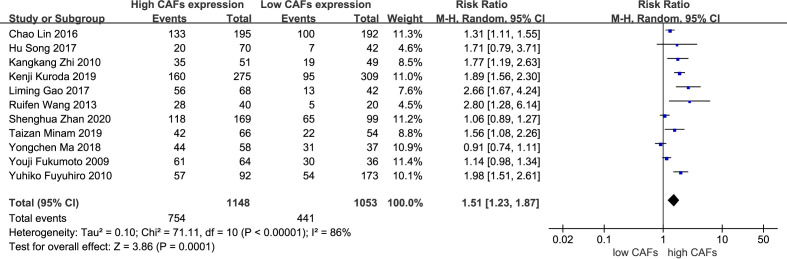
High expression of CAFs is associated with lymph node metastasis of gastric cancer. CAF, cancer-associated fibroblast; CI, confidence interval.

A total of 11 studies (12, 13, 15, 16, 18–21, 23–25) were included, involving 2,201 patients. The meta-analysis results showed that patients with high expression of CAFs were at a significantly increased risk of developing lymph node metastasis. The rate of lymph node metastasis in samples with high and low expression of CAFs was 65.4% and 41.9%, respectively. The RR of the study was 1.51 (95% CI: 1.23–1.87; P=0.0001) (**Figure 3**).

#### 3.3.3 High expression of CAFs was associated with serosal infiltration in GC

**Figure 4:**
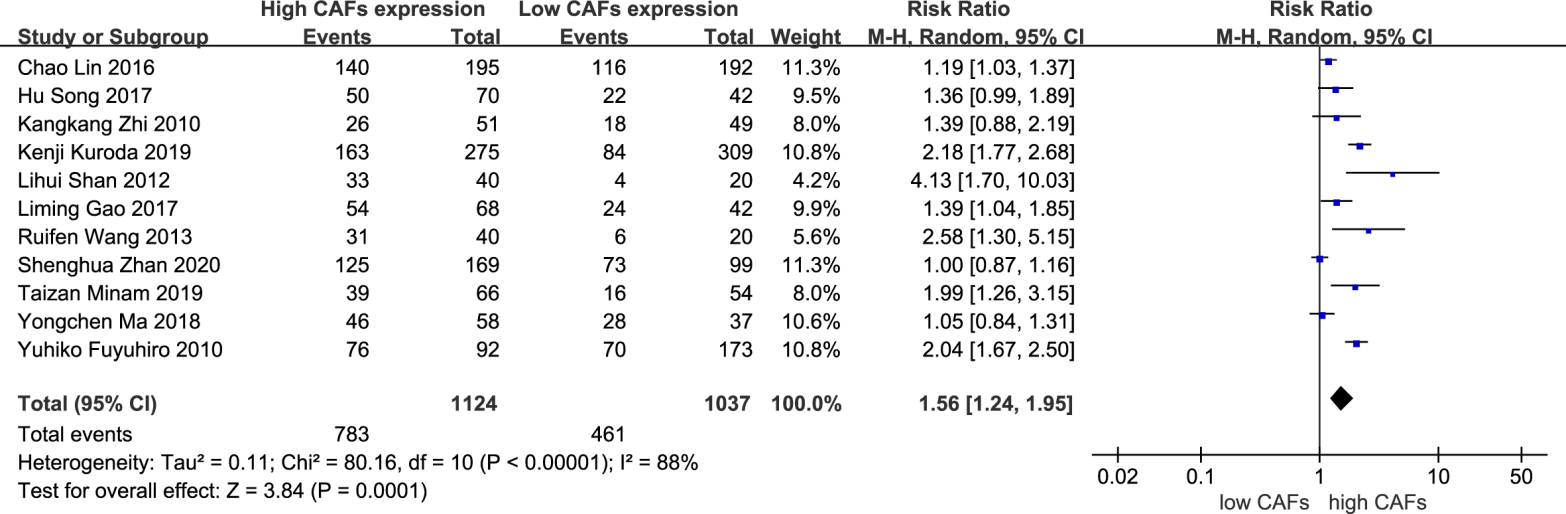
High expression of CAFs is associated with serosal infiltration in gastric cancer. CAF, cancer-associated fibroblast; CI, confidence interval.

A total of 11 studies (12, 13, 15–21, 23, 25) were included, involving 2,161 patients. The meta-analysis results showed that patients with high expression of CAFs were at a significantly increased risk of serosal infiltration. The rate of serosal infiltration in samples with high and low expression of CAFs was 69.7% and 44.5%, respectively. The RR of the study was 1.56 (95% CI: 1.24–1.95; P=0.0001) (**Figure 4**).

#### 3.3.4 High expression of CAFs was associated with diffuse and mixed GC in the Lauren classification

**Figure 5:**
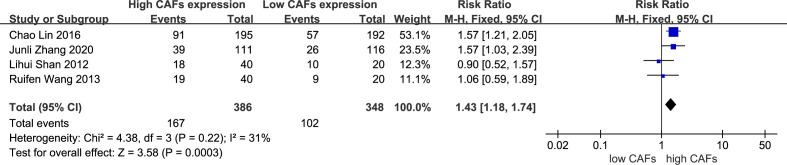
High expression of CAFs is associated with diffuse and mixed gastric cancer in the Lauren classification. CAF, cancer-associated fibroblast; CI, confidence interval.

Four studies (12, 14, 17, 19) were included, involving 734 patients. The meta-analysis results showed that patients with high expression of CAFs were at a significantly increased risk of diffuse and mixed GC in the Lauren classification. The rate of diffuse and mixed GC in the Lauren classification in samples with high and low expression of CAFs was 43.3% and 29.3%, respectively. The RR of the study was 1.43 (95% CI: 1.18–1.74; P=0.0003) (**Figure 5**).

#### 3.3.5 High expression of CAFs was associated with vascular invasion in GC

**Figure 6:**
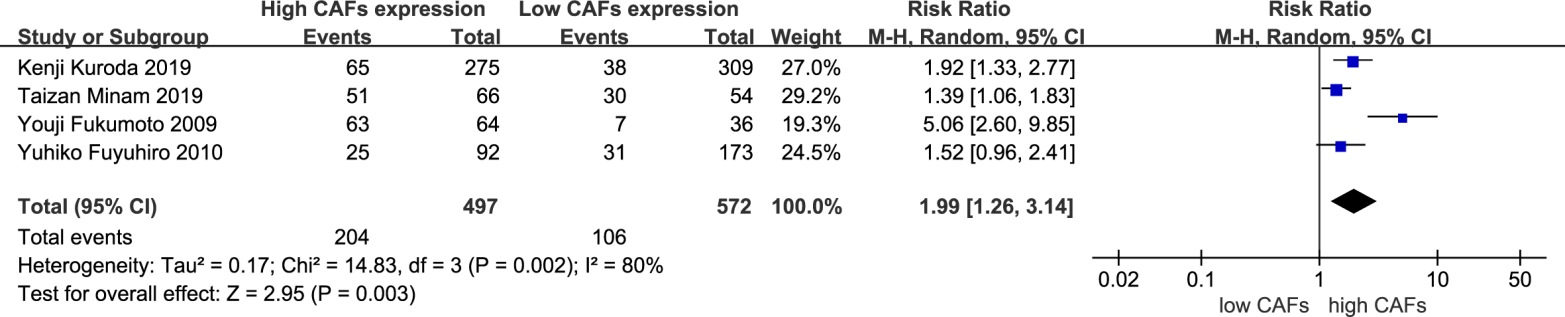
High expression of CAFs is associated with vascular invasion in gastric cancer. CAF, cancer-associated fibroblast; CI, confidence interval.

Four studies (16, 21, 24, 25) were included, involving 1,069 patients. The meta-analysis results showed that patients with high expression of CAFs were at a significantly increased risk of vascular invasion. The rate of vascular invasion in samples with high and low expression of CAFs was 41.0% and 18.5%, respectively. The RR of the study was 1.99 (95% CI: 1.26–3.14; P=0.003) (**Figure 6**).

#### 3.3.6 Correlation between CAFs, and differentiation and tumor size in GC

**Figure 7:**
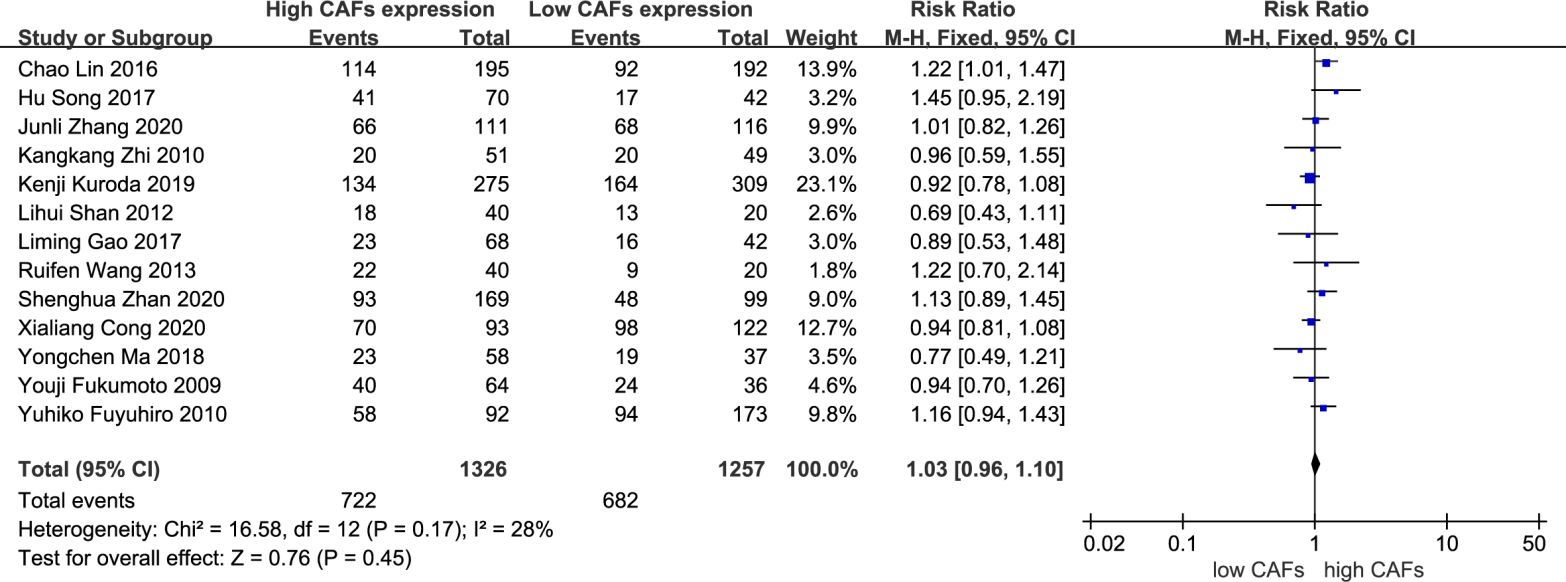
Correlation between CAFs and differentiation in gastric cancer. CAF, cancer-associated fibroblast; CI, confidence interval.

**Figure 8:**
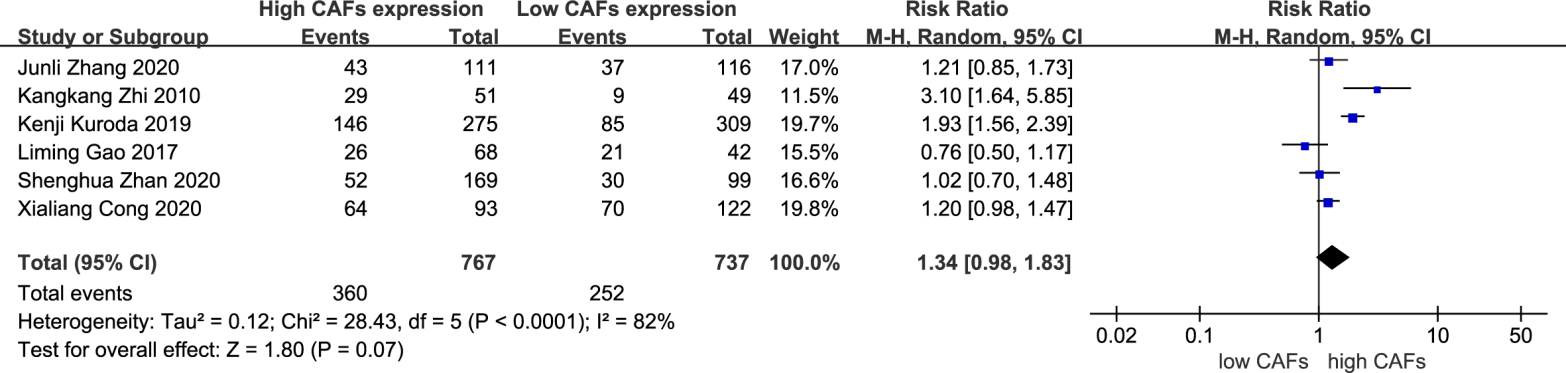
Correlation between CAFs and tumor size in gastric cancer. CAF, cancer-associated fibroblast; CI, confidence interval.

There was no significant correlation between CAF expression and poorly differentiated GC. Thirteen studies (12–20, 22–25) were included, involving 2,583 patients. The RR of the study was 1.03 (95% CI: 0.96–1.10; P=0.45) (**Figure 7**). In addition, CAF expression was not significantly correlated with GC with a tumor diameter >5 cm. Six studies were included (14–16, 18, 20, 22), involving 1,504 patients. The RR of the study was 1.34 (95% CI: 0.98–1.83; P=0.07) (**Figure 8**).

#### 3.3.7 CAFs as a prognostic factor for patients with GC

**Figure 9:**
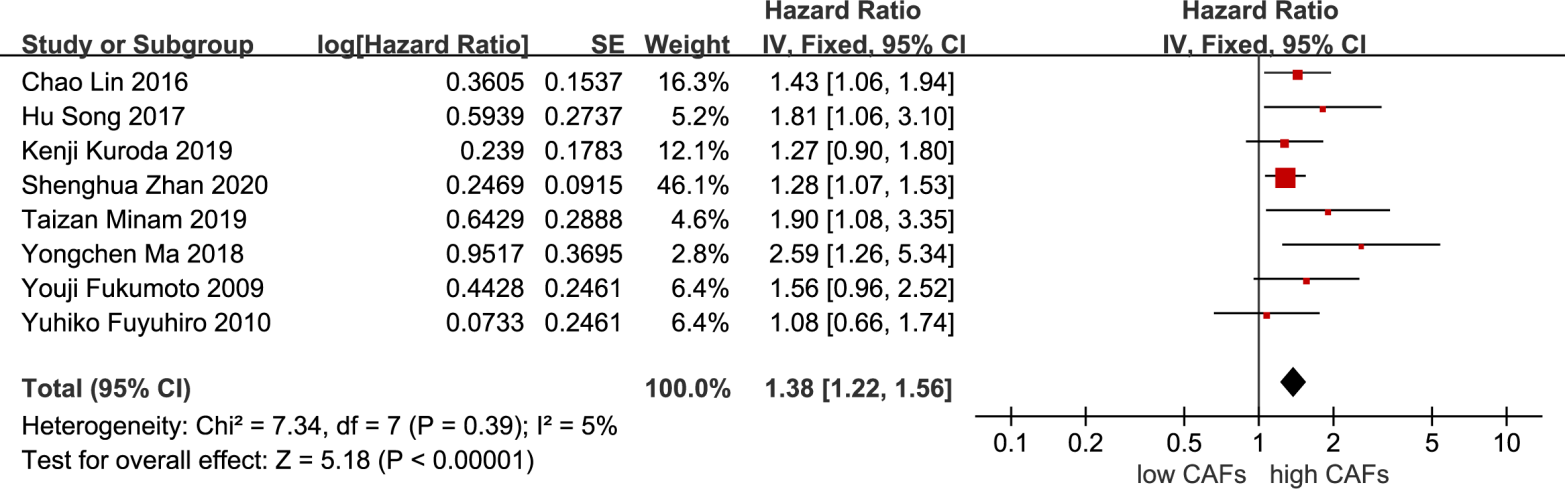
Association between CAFs and OS in gastric cancer. CAF, cancer-associated fibroblast; CI, confidence interval; OS overall survival; SE, standard error.

Eight studies (12, 13, 16, 20, 21, 23–25) were included. The meta-analysis results showed that high expression of CAFs was significantly associated with poor OS in patients with GC. The hazard ratio (HR) was 1.38 (95% CI: 1.22–1.56; P<0.00001) (**Figure 9**).

### 3.4 Subgroup analysis

Among the included studies, eight studies (12, 14–16, 20–22, 25) used α-SMA as a marker for CAF detection, while the remaining six studies (13, 17–19, 23, 24) used FAP. The specific role of CAFs identified by different markers in the development of GC is also controversial. Therefore, we performed a subgroup analysis based on these two different markers for the results of more than five included studies.

**Figure 10:**
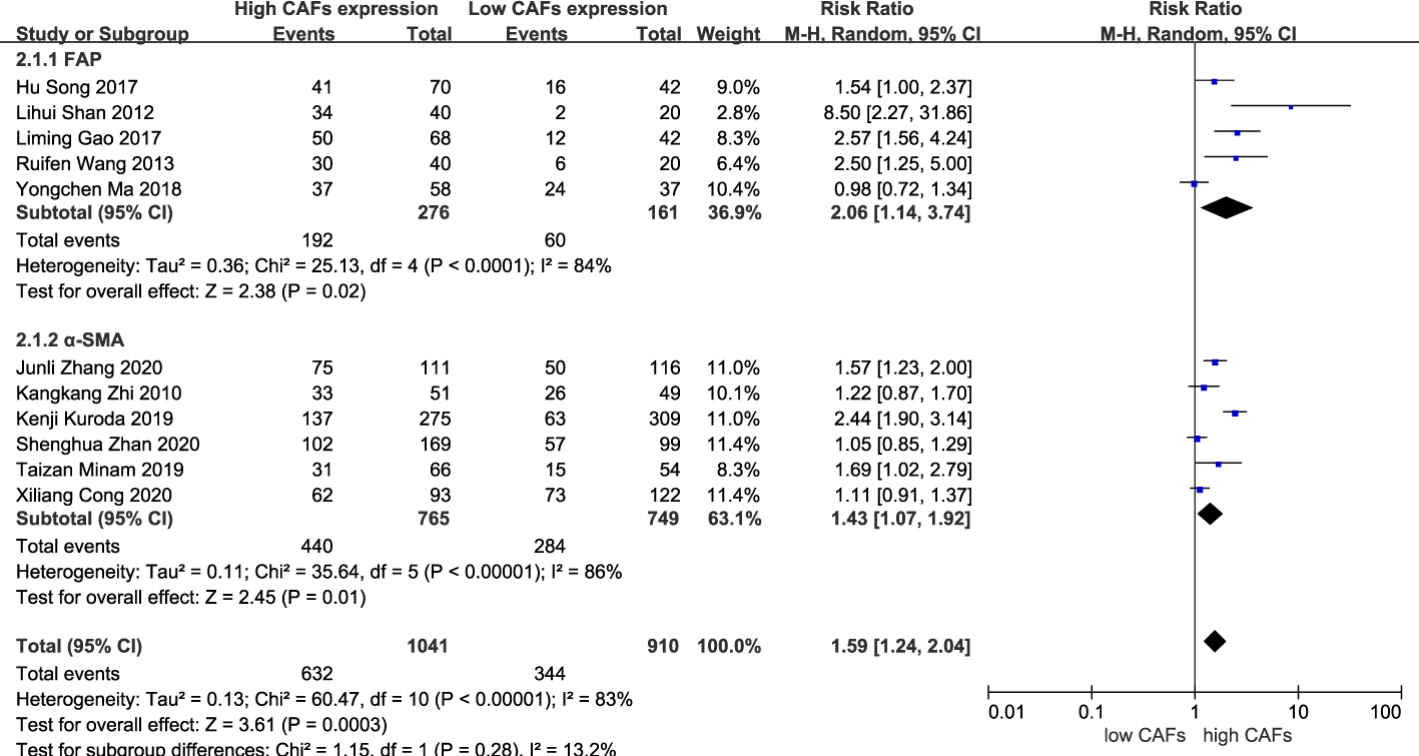
Subgroup analysis of high expression of CAFs and stage III–IV gastric cancer. CAF, cancer-associated fibroblast; CI, confidence interval.

**Figure 11:**
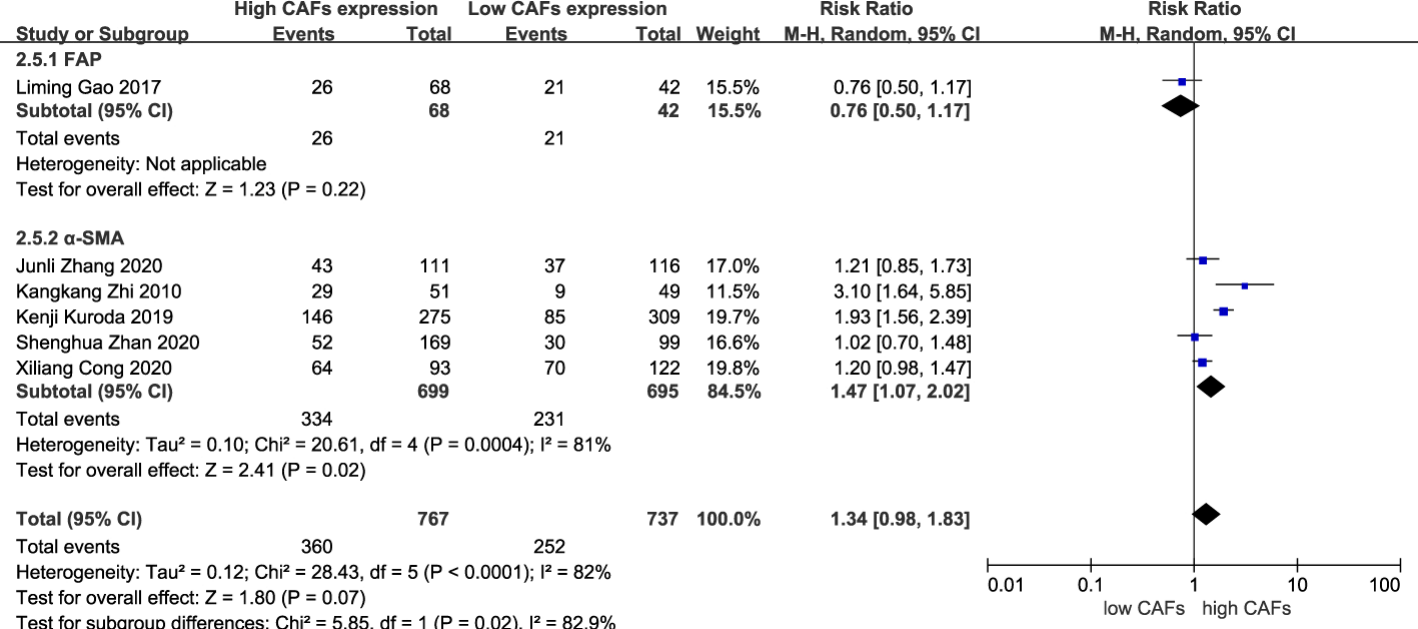
Subgroup analysis of high expression of CAFs and tumor size in gastric cancer. CAF, cancer-associated fibroblast; CI, confidence interval.

**Figure 12:**
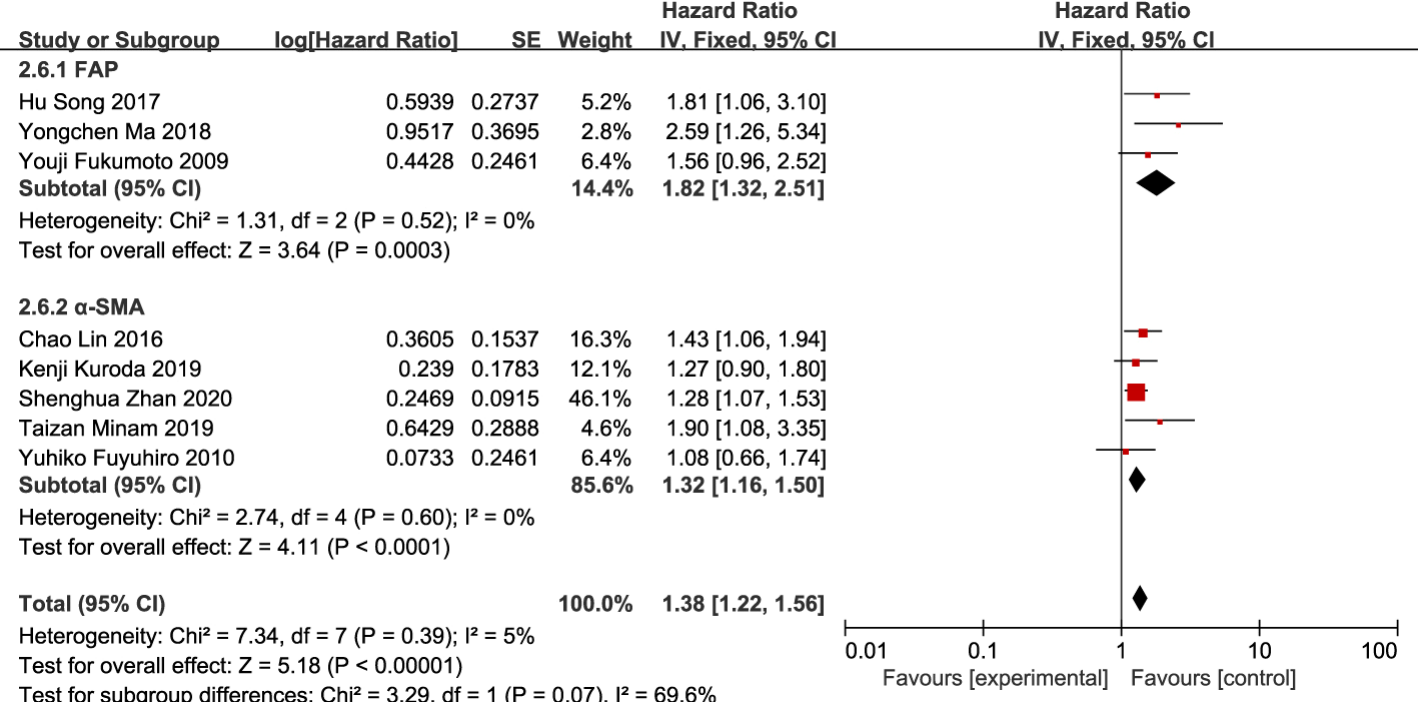
Subgroup analysis of high expression of CAFs and OS in gastric cancer. CAF, cancer-associated fibroblast; CI, confidence interval; OS overall survival; SE, standard error.

Subgroup analysis of high expression of CAFs and stage III–IV gastric cancer showed that FAP (RR, 2.06; 95% CI: 1.14–3.74; P=0.02)was more closely related to stage III-IV gastric cancer than α-SMA (RR, 1.43; 95% CI: 1.07–1.92; P=0.01) (**Figure 10**). Unexpectedly, although the result were not statistically significant in the meta-analysis of high expression of CAFs and tumor size in gastric cancer (P=0.07), in the subgroup analysis, we found a significant association between α-SMA and tumor diameter >5 cm (RR, 1.47; 95% CI: 1.07–2.02; P=0.02) (**Figure 11**). In addition, the FAP subgroup was also more strongly associated with poor OS (RR, 1.82; 95% CI: 1.32–2.51; P=0.0003) (**Figure 12**).

### 3.5 Sensitivity analyses

We conducted this meta-analysis after eliminating studies one by one, and the results showed no significant changes, indicating that the stability of the results was good.

### 3.6 Publication bias

The publication bias of the outcomes with more than 10 studies was assessed using visual examination of funnel plots and the Egger weighted regression statistic. The graph is shown in the **Supplementary File**. No significant publication bias was indicated.

## 4 Discussion

Cancer is not limited to the presence of malignant tumor cells. It is characterized by a fundamental imbalance of the entire cell environment, termed TME, which is a complex dynamic system composed of cellular and non-cellular components (26). CAFs are one of the most important components in the TME and play an essential role in the occurrence and development of tumors. In recent years, an increasing number of studies have focused on tumor-associated fibroblasts and reported their roles in tumor proliferation, invasion, metastasis, drug resistance, etc. (27). Numerous studies investigated targeted therapies for CAFs (28). A meta-analysis of the association of CAFs with the prognostic characteristics of oral squamous cell carcinoma and head and neck squamous cell carcinoma was previously conducted, revealing significant correlations (29, 30). The correlation between CAFs and the prognosis of gastrointestinal tumors was also analyzed. However, due to the small number of GC studies included in this meta-analysis, the correlations between individual GC pathological features and CAFs were not analyzed (31).

This study systematically analyzed the relationship between CAFs and clinicopathological characteristics and prognosis of GC. A total of 14 studies were included, with sample sizes ranging from 60 to 594 patients. The present study confirmed that high expression of CAFs was closely associated with pathological indicators related to advanced GC (e.g., stage, lymph node metastasis, and vascular metastasis), suggesting that CAFs play a key role in GC invasion and metastasis. Several basic research studies confirmed that CAFs promote GC invasion and metastasis by inducing epithelial–mesenchymal transition (EMT), extracellular matrix (ECM) remodeling, and tumor angiogenesis. EMT alters the morphology of tumor cells from tightly arranged epithelial cells to loosely structured mesenchymal cells. This effect weakens the adhesion between tumor cells and enhances their motility, thus facilitating detachment from the primary site and transfer to other sites. Studies have confirmed that inflammatory cytokines, such as interleukin 6 (IL-6), IL-11, and IL-33, secreted by CAFs promote EMT through downstream signaling pathways, such as Janus kinase and signal transducer and activator of transcription and mitogen-activated protein kinase/extracellular signal-regulated kinase pathways. Moreover, they promote the migration and invasion of GC cells, resulting in peritoneal dissemination of GC (32–35). Downregulation of mirNA-214 in CAFs induces EMT and promotes the migration and invasion of GC cells (36). In ECM remodeling, CAFs can degrade the ECM by expressing matrix metalloproteinases and collagenase, and change its structure and hardness through the release of transforming growth factor-β (TGF-β) (37). CAFs can also increase the hardness of the ECM by regulating factors related to cytoskeleton formation, thereby promoting tumor invasion and metastasis. Recent studies have found that hyaluronan and proteoglycan link protein 1, the most significantly upregulated gene in CAFs of GC, promotes invasion and metastasis through TGF-β-mediated ECM remodeling (38). Regarding tumor angiogenesis, blood vessels are the main channel of tumor cell metastasis and the main source of nutrients for tumor cells. The formation of tumor blood vessels is a sign of malignancy. CAFs promote tumor angiogenesis by secreting cytokines, such as vascular endothelial growth factor, platelet-derived growth factor, and TGF-β (39). Studies have confirmed that CAF-derived hepatocyte growth factor promotes angiogenesis, vascular mimicry, and mosaic vascular formation through the phosphatidylinositol 3 kinase/protein kinase B and extracellular regulated kinase 1/2 signaling pathways (40).

The subanalysis performed to determine the degree of differentiation in GC included the largest number of studies and cases in this investigation. There were no significant differences or heterogeneity among the studies. Unlike in head and neck squamous cell carcinoma, CAFs were not significantly correlated with the degree of differentiation in GC. Further basic research is warranted to confirm the relationship between differentiation and CAFs in GC. In addition, some indicators (e.g., microsatellite instability, HER2, PD-L1, Epstein–Barr virus, KI-67, etc.) cannot be comprehensively analyzed because the number of studies focusing on these indicators is currently insufficient; hence, it is important to further investigate the correlation between indicators in the future.

Although the present study demonstrated that high expression of CAFs is closely associated with the traditional pathological indicators related to poor prognosis in GC, CAFs did not show significant advantages over those characteristics and prognostic markers. Traditionally, CAFs have been broadly differentiated based on their morphology and specific markers. However, in recent years, the progress of single-cell omics technology has enabled researchers to further distinguish various types of CAFs and conduct more detailed studies on their specific roles (41–44). Through single-cell sequencing of GC tissues and adjacent mucosal samples, Li et al. (45) identified four subsets of CAFs with different properties, namely myofibroblastic CAFs, pericytic CAFs, inflammatory CAFs, and ECM CAFs. Inflammatory CAFs and ECM CAFs show enhanced invasive activity and mobilize surrounding immune cells to construct a tumor-friendly microenvironment, which is associated with poor prognosis of GC. Recently, a pan-cancer single-cell analysis reveal the heterogeneity of CAF and revealed their different activation pathways and roles (46). The article proposed that a high proportion of FAP+ CAFs was significantly associated with poor OS, which was similar to the conclusion obtained in our study. The article also proposed that α-SMA+ CAFs are closely related to angiogenesis, but there were not enough samples in the studies we included. So, additional studies are needed to examine the relationship between CAF heterogeneity and the pathological features and prognosis of GC.

This study had some limitations. Firstly, all studies included in this meta-analysis were conducted in China (n=10) and Japan (n=4). Hence, there was a lack of studies from other countries. This may be related to the global distribution of GC. Although GC is the fifth most malignant type of cancer worldwide, its incidence is low in European and American countries. Secondly, most studies did not adopt uniform standards for the determination of CAF expression, and the distinction between high and low CAF expression was not completely consistent. Thus, the results of this meta-analysis may be biased to some extent.

## 5 Conclusion

This study analyzed the relationship between CAF expression and clinicopathological indicators and prognosis of GC. The results showed that high expression of CAFs was closely associated with the traditional pathological indicators related to poor prognosis of GC, and may be valuable predictor of poor prognosis in this setting. This may provide new directions for research on the related mechanism and targeted therapy of GC. However, more high-quality studies are warranted to verify the above conclusions.

## Data availability statement

The original contributions presented in the study are included in the article/**Supplementary Material**. Further inquiries can be directed to the corresponding author.

## Author contributions

Concept and design: JW. Acquisition of data: JW and MW. Statistical analysis: JW and MW. Interpretation of data: all authors. Writing of the original draft of the manuscript: JW and MW. Review and editing of the manuscript: all authors. All authors contributed to the article and approved the submitted version.
