# Supplementary material for: Cancer-associated fibroblasts, and clinicopathological characteristics and prognosis of gastric cancer: A systematic review and meta-analysis
Source: Front Oncol. 2023 Feb 17;13:1048922. doi: 10.3389/fonc.2023.1048922 (PMC9981791; doi:10.3389/fonc.2023.1048922)
Supplement: Supplementary file 1 [file Table_1.pdf]

Publication Bias

3.3.1 High expression of CAFs was associated with stage III–IV GC

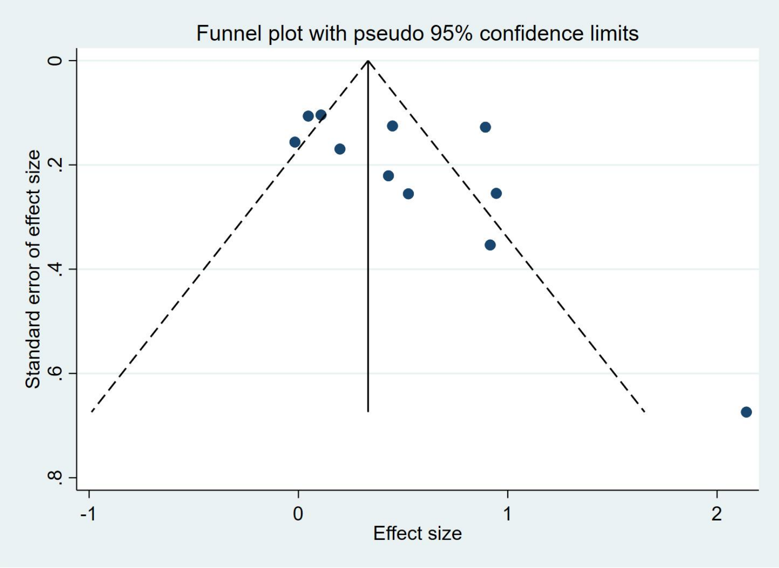

Number of studies = **11** Root MSE = **2.056**

Std_Eff	Coef.	Std. Err.	t	P>|t|	[95% Conf.	Interval]
slope	**-.0967985**	**.2411559**	**-0.40**	**0.697**	**-.642331**	**.448734**
bias	**2.980351**	**1.529347**	**1.95**	**0.083**	**-.4792719**	**6.439974**

Test of H0: no small-study effects P = **0.083**

3.3.2 High expression of CAFs was associated with lymph node metastasis in GC

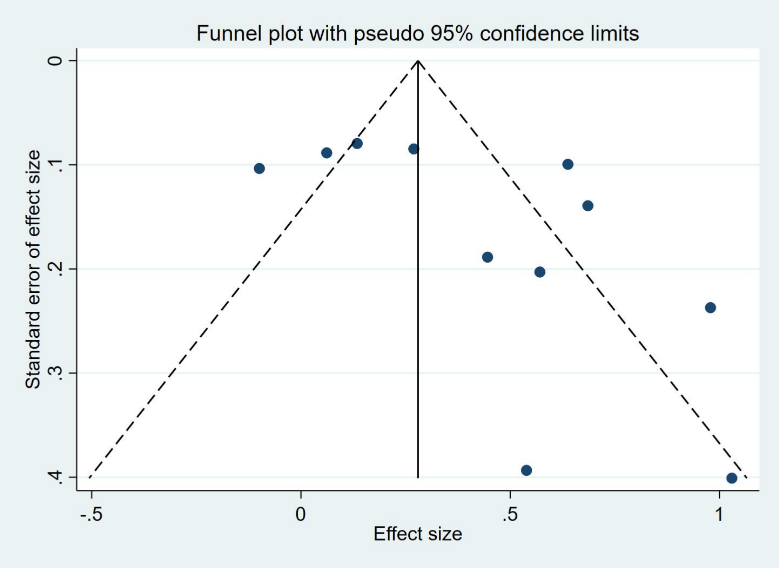

Number of studies = **11** Root MSE = **2.647**

Std_Eff	Coef.	Std. Err.	t	P>|t|	[95% Conf.	Interval]
slope	**.3143762**	**.3047796**	**1.03**	**0.329**	**-.3750832**	**1.003835**
bias	**.6638385**	**2.021578**	**0.33**	**0.750**	**-3.90929**	**5.236967**

Test of H0: no small-study effects P = **0.750**

3.3.3 High expression of CAFs was associated with serosal infiltration in GC

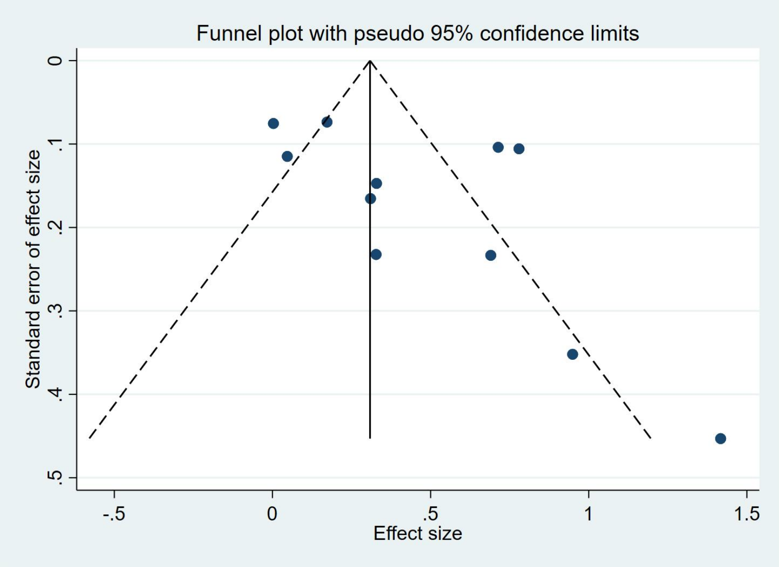

Number of studies = **11** Root MSE = **2.217**

Std_Eff	Coef.	Std. Err.	t	P>|t|	[95% Conf.	Interval]
slope	**.197057**	**.2700955**	**0.73**	**0.484**	**-.4139416**	**.8080556**
bias	**1.99294**	**1.763867**	**1.13**	**0.288**	**-1.997204**	**5.983084**

Test of H0: no small-study effects P = **0.288**

3.3.6 Correlation between CAFs, and differentiation and tumor size in GC

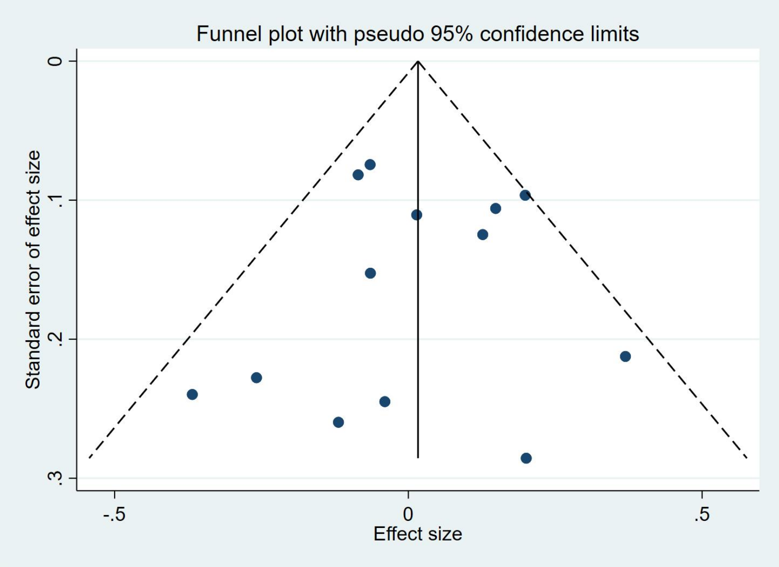

Number of studies = **13** Root MSE = **2.308**

Std_Eff	Coef.	Std. Err.	t	P>|t|	[95% Conf.	Interval]
slope	**.3114613**	**.2974473**	**1.05**	**0.318**	**-.3432157**	**.9661384**
bias	**-2.122056**	**2.132273**	**-1.00**	**0.341**	**-6.815158**	**2.571045**

Test of H0: no small-study effects P = **0.341**
