# Supplementary material for: Cancer-associated fibroblasts, and clinicopathological characteristics and prognosis of gastric cancer: A systematic review and meta-analysis
Source: Front Oncol. 2023 Feb 17;13:1048922. doi: 10.3389/fonc.2023.1048922 (PMC9981791; doi:10.3389/fonc.2023.1048922)
Supplement: Supplementary file 2 [file Table_2.docx]

**Pubmed**

#1. "Stomach Neoplasms"[Mesh]

#2. stomach[Title/Abstract] OR gastric[Title/Abstract]

#3. neoplasm*[Title/Abstract] OR cancer*[Title/Abstract] OR tumor*[Title/Abstract] OR carcinoma*[Title/Abstract] OR adenocarcinoma*[Title/Abstract]

#4. #2 AND #3

#5. #1 OR #4

#6. "Cancer-Associated Fibroblasts"[Mesh]

#7.cancer-associated[Title/Abstract] OR tumor-associated[Title/Abstract] OR carcinoma-associated[Title/Abstract] OR stomach-associated[Title/Abstract] OR gastric-associated[Title/Abstract]

#8. associated[Title/Abstract]

#9. #3 AND #8

#10. fibroblast*[Title/Abstract]

#11. (#7 OR #9) AND #10

#12.CAF*[Title/Abstract] OR myofibroblast*[Title/Abstract] OR activated fibroblast*[Title/Abstract]

#13. fibroblast activation protein[Title/Abstract] OR α-smooth muscle actin[Title/Abstract]

#14. #6 OR #11 OR #12 OR #13

#15. #5 AND #14

**Embase**

#1. 'stomach cancer'/exp

#2. stomach:ti,ab,kw OR gastric:ti,ab,kw

#3. neoplasm*:ti,ab,kw OR cancer*:ti,ab,kw OR tumor*:ti,ab,kw OR carcinoma*:ti,ab,kw OR

adenocarcinoma*:ti,ab,kw

#4. #2 AND #3

#5. #1 OR #4

#6. 'cancer associated fibroblast'/exp

#7. 'cancer associated':ti,ab,kw OR 'tumor associated':ti,ab,kw OR 'carcinoma associated':ti,ab,kw OR 'stomach associated':ti,ab,kw OR 'gastric associated':ti,ab,kw

#8. associated:ti,ab,kw

#9. #3 AND #8

#10. fibroblast*:ti,ab,kw

#11. (#7 OR #9) AND #10

#12. caf*:ti,ab,kw OR myofibroblast*:ti,ab,kw OR 'activated fibroblast*':ti,ab,kw

#13. 'fibroblast activation protein':ti,ab,kw OR 'α-smooth muscle actin':ti,ab,kw

#14. #6 OR #11 OR #12 OR #13

#15. #5 AND #14

**Web of science**

1: TS=(stomach OR gastric)

2: TS= (neoplasm* OR cancer* OR tumor* OR carcinoma* OR adenocarcinoma*)

3: #1 AND #2

4: TS=(cancer-associated OR tumor-associated OR carcinoma-associated OR stomach-associated OR gastric-associated)

5: TS=(associated)

6: #2 AND #5

7: TS=(fibroblast*)

8: (#4 OR #6) AND #7

9: TS=(CAF* OR myofibroblast* OR activated fibroblast*)

10: TS=(fibroblast activation protein OR α-smooth muscle actin)

11: #8 OR #9 OR #10

12: #3 AND #11

**The Cochrane library**

#1. MeSH descriptor: [Stomach Neoplasms] explode all trees

#2. (stomach or gastric):ti,ab,kw

#3. (neoplasm* or cancer* or tumor* or carcinoma* or adenocarcinoma*):ti,ab,kw

#4. #2 and #3

#5. #1 or #4

#6. MeSH descriptor: [Cancer-Associated Fibroblasts] explode all trees

#7. (cancer-associated or tumor-associated or carcinoma-associated or stomach-associated or gastric-associated):ti,ab,kw

#8. (associated):ti,ab,kw

#9. #3 and #8

#10. (fibroblast*):ti,ab,kw

#11. (#7 or #9) and #10

#12. (CAF* or myofibroblast* or activated fibroblast*):ti,ab,kw

#13. (CAF* or myofibroblast* or activated fibroblast*):ti,ab,kw

#14. #6 OR #11 OR #12 OR #13

#15. #5 AND #14
